# Functional Characterization of a Novel Frameshift Mutation in the C-terminus of the Nav1.5 Channel Underlying a Brugada Syndrome with Variable Expression in a Spanish Family

**DOI:** 10.1371/journal.pone.0081493

**Published:** 2013-11-25

**Authors:** Pablo Dolz-Gaitón, Mercedes Núñez, Lucía Núñez, Adriana Barana, Irene Amorós, Marcos Matamoros, Marta Pérez-Hernández, Marta González de la Fuente, Miguel Álvarez-López, Rosa Macías-Ruiz, Luis Tercedor-Sánchez, Juan Jiménez-Jáimez, Eva Delpón, Ricardo Caballero, Juan Tamargo

**Affiliations:** 1 Department of Pharmacology, School of Medicine, Universidad Complutense de Madrid, Madrid, Spain; 2 Instituto de Investigación Sanitaria Gregorio Marañón, School of Medicine, Universidad Complutense, Madrid, Spain; 3 Instituto de Investigación Sanitaria Hospital Clínico San Carlos, School of Medicine, Universidad Complutense, Madrid, Spain; 4 Complejo Hospitalario Universitario de A Coruña and Instituto de Ciencias de la Salud, Universidad de A Coruña, A Coruña, Spain; 5 Arrhytmias Unit, Cardiology Department, Hospital Universitario Virgen de las Nieves, Granada, Spain; University of Milan, Italy

## Abstract

**Introduction:**

We functionally analyzed a frameshift mutation in the *SCN5A* gene encoding cardiac Na^+^ channels (Nav1.5) found in a proband with repeated episodes of ventricular fibrillation who presented bradycardia and paroxysmal atrial fibrillation. Seven relatives also carry the mutation and showed a Brugada syndrome with an incomplete and variable expression. The mutation (p.D1816VfsX7) resulted in a severe truncation (201 residues) of the Nav1.5 C-terminus.

**Methods and Results:**

Wild-type (WT) and mutated Nav1.5 channels together with hNavβ1 were expressed in CHO cells and currents were recorded at room temperature using the whole-cell patch-clamp. Expression of p.D1816VfsX7 alone resulted in a marked reduction (≈90%) in peak Na^+^ current density compared with WT channels. Peak current density generated by p.D1816VfsX7+WT was ≈50% of that generated by WT channels. p.D1816VfsX7 positively shifted activation and inactivation curves, leading to a significant reduction of the window current. The mutation accelerated current activation and reactivation kinetics and increased the fraction of channels developing slow inactivation with prolonged depolarizations. However, late I_Na_ was not modified by the mutation. p.D1816VfsX7 produced a marked reduction of channel trafficking toward the membrane that was not restored by decreasing incubation temperature during cell culture or by incubation with 300 μM mexiletine and 5 mM 4-phenylbutirate.

**Conclusion:**

Despite a severe truncation of the C-terminus, the resulting mutated channels generate currents, albeit with reduced amplitude and altered biophysical properties, confirming the key role of the C-terminal domain in the expression and function of the cardiac Na^+^ channel.

## Introduction

*SCN5A* gene encodes the α-subunit of cardiac voltage-gated Na^+^ channels (Nav1.5), which generate the inward sodium current (I_Na_) that is critical for the genesis and propagation of action potentials and, in turn, determines cardiac excitability and conduction velocity of the electrical impulse within the heart [1,2]. Nav1.5 comprises 4 homologous domains, DI to DIV, each of which contains 6 transmembrane helices (S1 to S6) with intracellular N- and C-terminal domains [3]. Mutations in *SCN5A* have been associated with several arrhythmogenic diseases. Gain-of-function mutations leading to an increased late I_Na_ (I_Na,L_) cause long QT syndrome type 3 (LQT3), whereas loss-of-function mutations resulting in a decreased peak I_Na_ may cause a variety of arrhythmogenic syndromes such as Brugada syndrome [characterized by the elevation of the ST segment in the right precordial leads of the ECG (BrS)], progressive cardiac conduction disease, sick sinus syndrome, atrial fibrillation (AF) [4], and sudden infant death syndrome [2-4]. Furthermore, loss-of-function Nav1.5 mutations have been described in patients with idiopathic ventricular fibrillation (IVF), an uncommon and lethal condition which presents itself as syncope or sudden cardiac death in young people with normal hearts and without electrophysiological manifestations of inherited arrhythmogenic syndromes [5-7]. Therefore, Nav1.5 mutations can render a broad spectrum of inherited cardiac arrhythmias. Furthermore, some *SCN5A* mutations can lead to complex diseases associating different phenotypic traits such as, for instance, bradycardia, conduction disease, LQT3, and BrS, i.e., the so-called overlap syndromes [8].

C-terminus of Nav1.5 channels which comprises 243 residues plays an important role in regulating both channel gating and membrane expression. The predicted structure of the first half of the C-terminal domain consists of six helices (H1-H6) while the second half is not structured. It has been proposed that C-terminus helices participate in the control of inactivation through stabilization of the closed gate [9,10]. Furthermore, C-terminal domain bears several regions critical for protein-protein interaction, particularly the PDZ binding domain, which, in turn, are critical for channel trafficking and surface expression. A huge amount of disease-causing mutations have been identified in transmembrane segments of Nav1.5. However, mutations in the C-terminal domain are less frequent and only a few of them have been studied functionally [1-3,11-13].

Here we report a novel heterozygous frameshift mutation in *SCN5A* that results in a severe truncation of the C-terminal domain found in a proband with repeated episodes of ventricular fibrillation who presented bradycardia and paroxysmal AF. Heterologous expression of the mutation resulted in a marked decrease of peak I_Na_ density mainly caused by a reduced channel trafficking toward the plasma membrane and in severe alterations in channel activation and inactivation. Interestingly, unlike other C-terminal domain truncating mutations [11], the mutation here presented did not modify I_Na,L_ suggesting that deletions of different lengths can differentially affect gating properties of the variants. This fact is important since these differences contribute to determining the phenotype of the carriers. Furthermore, our results suggest that this *SCN5A* mutation can result in multiple rhythm disturbances within the same family, presenting with extensive variability in type and severity of symptoms, including BrS, conduction disease, and AF. 

## Methods

### Genetic testing

The study was approved by the Investigation Committee of the Hospital Universitario Virgen de las Nieves and conforms to the principles outlined in the Declaration of Helsinki. Written informed consent was obtained for the genetic screening test of the proband and all the relatives studied. Genomic DNA was isolated from white blood cells by conventional methods [14,15]. The whole codifying sequence and the flanking intronic regions of *KCNQ1*, *KCNH2*, *KCNJ2, SCN5A*, *KCNE1*, and *KCNE2* genes were amplified by polymerase chain reaction and directly sequenced. *SCN5A* (hH1 clone) mutation was introduced using the QuikChange Site-Directed Mutagenesis kit (Stratagene, USA) and confirmed by direct DNA sequencing [15].

### Electrophysiological analysis

Wild-type (WT) and mutated Nav1.5 channels (0.8 µg) together with the ancillary subunit hNavβ1 (0.8 µg) were transiently transfected in CHO cells by using Fugene 6 (Roche Diagnostics, Switzerland). Currents were recorded at room temperature using the whole-cell patch-clamp configuration following previously described methods [15]. Recording pipettes were pulled from 1.0 mm o.d. borosilicate capillary tubes (GD1, Narishige Co., Ltd, Japan) using a programmable patch micropipette puller (Model P-2000 Brown-Flaming, Sutter Instruments Co., USA) and were heat-polished with a microforge (Model MF-830, Narishige). Micropipette resistance ranged from 0.5 to 1.5 MΩ when filled with the internal solution and immersed in the external solution. In all experiments, series resistance was compensated manually by using the series resistance compensation unit of the Axopatch-200B amplifier, and usually ≥80% compensation was achieved. Mean peak maximum Nav1.5 current (I_Nav1.5_) amplitude, uncompensated access resistance, and capacitance averaged 3.0±0.4 nA, 1.6±0.2 MΩ, and 7.8±0.7 pF (n=69), respectively. Thus, under our experimental conditions no significant voltage errors (<5 mV) due to series resistance were expected with the micropipettes used. Currents were filtered at half the sampling frequency and stored on the hard disk of a computer for subsequent analysis by using p.CLAMP9 software (Molecular Devices, USA). When WT and mutant Nav1.5 channels were coexpressed, a 0.5:0.5 ratio was used. To minimize the influence of the expression variability of transiently transfected mammalian cell lines, each construct was tested in a large number of cells obtained from at least three different transfection batches. Cells were perfused with an external solution containing (mM): NaCl 136, KCl 4, CaCl_2_ 1.8, MgCl_2_ 1, HEPES 10, and glucose 10 (pH 7.4 with NaOH). The internal solution contained (mM): NaF 10, CsF 110, CsCl 20, HEPES 10, and EGTA 10 (pH 7.35 with CsOH). In a separate group of experiments, I_Nav1.5_ currents generated by WT and mutant channels were recorded in the absence of F^-^ as previously described by Ruan et al [16]. In this group of experiments the composition of the external solution was (mM): NaCl 130, CaCl_2_ 2, CsCl 5, MgCl_2_ 1.2, HEPES 10, and glucose 5 (pH=7.4 with CsOH), while that of the internal solution was (mM): aspartic acid 50, CsCl 60, Na_2_-ATP 5, EGTA 11, HEPES 10, CaCl_2_ 1, and MgCl_2_ 1 (pH=7.4 with CsOH). 

The protocol to obtain I_Nav1.5_-voltage (I-V) curves consisted of 50 ms pulses in 5 mV increments from –120 mV to potentials between −80 and +50 mV. To minimize the contribution of time-dependent shifts of channel availability, all data were collected 20 min after establishing the whole-cell configuration. Activation curves were constructed plotting the normalized peak conductance as a function of the membrane potential. The conductance was estimated by the equation [G=I/(V_m_-E_rev_)], where G is the conductance at the test potential V_m_, I represents the peak maximum current at V_m_, and E_rev_ is the reversal potential. To determine the E_rev_, the current density-voltage relationships were fitted to a function of the form [I=(V_m_-E_rev_)*G_max_*(1+exp[V_m_-V_h_]/*k*)^-1^], where I is the peak current elicited at the test potential V_m_, G_max_ is the maximum conductance, and *k* is the slope factor. To obtain the inactivation curves, a two-step protocol was used. The first 500-ms conditioning pulse from –120 mV to potentials between –140 and –20 mV was followed by a 20-ms test pulse to –20 mV. Inactivation curves were constructed by plotting the current amplitude obtained with the test pulse normalized to the largest peak current, as a function of the voltage command of the conditioning pulse. The fit of a Boltzmann function to the data yielded the midpoint (V_h_) and the slope (k) of the activation and inactivation curves. We also analyzed the effect of the mutation on the Na_v_1.5 window current. The overlap of steady-state activation and steady-state inactivation curves of Na^+^ channels identifies a range of voltages (i.e., window) where the channels have a small probability of being partially but not fully inactivated [17]. The probability of being within this window was calculated from the product of activation and steady-state inactivation parameters through the following equation: Probability = (1/{1 + exp[(V_hact_ − V)/k_act_]} × ((1 − C)/{1 + exp[(V − V_hinact_)/k_inact_]} + C) as previously described [18]. 

### Membrane trafficking

To identify trafficking defects induced by the mutation, Na^+^ channels (hH1 clone) conjugated to green fluorescent protein (GFP) were localized by confocal microscopy [19]. In this construct, GFP was inserted to the C-terminal domain of the Nav1.5 channel as previously described [20]. CHO cells were transfected with the constructs and collected 48 h after transfection following the procedures described above. In a group of experiments, density, and voltage-dependence of activation and inactivation of the currents generated by Nav1.5 WT channels conjugated or not with GFP were compared. Currents were recorded as described above. For confocal microscopy experiments, cells were seeded on fibronectine-coated coverslips and allowed to attach for 30 min. Afterwards, they were washed with phosphate buffered saline (PBS) and fixed with 4% paraformaldehyde for 10 min. Then, they were washed again and incubated for 10 min with a wheat germ agglutinin Alexa Fluor 647 conjugate (Invitrogen, UK), a fluorescent plasma membrane dye. Finally, the coverslips were washed three times with PBS and mounted using the Prolong (Invitrogen) antifading mounting medium. Confocal microscopy was performed with a Leica TCS-SP5 AOBS confocal microscope (Mannheim, Germany) with 20x and 63x oil immersion optics. Laser lines at 488 nm and 633 nm for excitation of GFP and Alexa Fluor were provided by an Argon laser and a Helio-Neon laser, respectively. Detection ranges were set to eliminate crosstalk between fluorophores. The XY frame was set to 512×512 pixels, and laser intensity was set to 6% power. The z axis was changed in ≈0.50 μm increments by computer control through the entire volume of the cell. Colocalization of channels and membrane marker was quantified using BioImageXD software for calculation of the percentage of colocalized voxels. 

### Statistical analysis

Results are expressed as mean±SEM. Unpaired *t*-test or one-way ANOVA followed by Newman-Keuls test were used to assess statistical significance where appropriate. A value of P<0.05 was considered significant.

## Results

### Case description and genetic analysis

**Figure 1:**
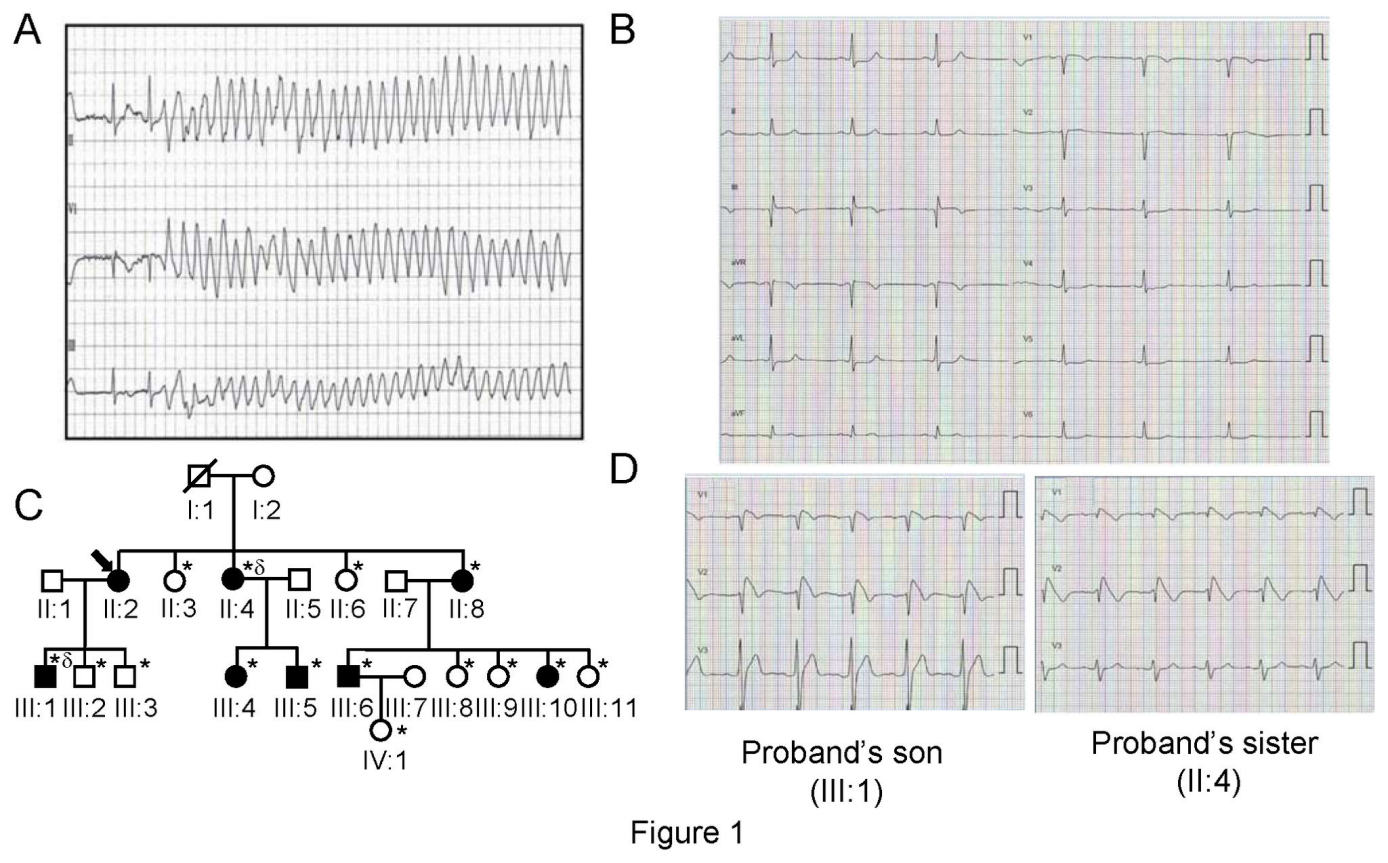
Clinical characterization and pedigree of the proband. **A**. ECG of the proband at hospital admission with an episode of ventricular fibrillation initiated by a premature ventricular contraction (paper speed 25 mm/s). Reprinted from [21] under a CC BY license, with permission from Elsevier original copyright (2011). **B**. 12-Lead ECG of the proband after flecainide continuous infusion at 2 mg/kg for 10 min (paper speed 25 mm/s). **C**. Pedigree of the affected family. Circles and squares represent female and male subjects, respectively. White and black symbols represent unaffected and affected individuals, respectively. The arrow denotes the proband. Diagonal lines indicate deceased subjects. The asterisk denotes proband`s relatives subjected to genetic and electrophysiological analyses (including flecainide test). Patient named in the figure as III.4 was excluded from the pharmacological test and ECG analysis because she had suffered Tetrallogy of Fallot that had been corrected in childhood, although she is a mutation carrier. ^*δ*^ denotes proband`s relatives who displayed type 1 BrS pattern after flecainide challenge. **D**. ECG of the proband´s son (top) and sister (bottom) after flecainide challenge.

The proband, a 53-year old woman with no family history of cardiac disease or sudden death, was asymptomatic until she was admitted to the hospital after a syncopal episode. Electrocardiographic monitoring showed the presence of polymorphic ventricular tachycardia with spontaneous cessation and frequent monomorphic premature ventricular complexes with left bundle branch block and left superior axis morphology and short coupling intervals, which triggered repeated episodes of ventricular fibrillation (Figure 1A). These episodes were resistant to treatment with β-blockers, amiodarone, procainamide, and isoproterenol, requiring 20 defibrillations over a period of 72 h [21]. Afterwards, an internal cardioverter defibrillator was implanted and since then she has been under regular follow up with no more subsequent arrhythmic events up to now. 

Interestingly, the proband presented sinus bradycardia (45-50 bpm) on basal status and under flecainide (Figure 1B) and also paroxysmal AF. Structural heart disease and other possible cardiac conditions were excluded by echocardiograghy, cardiac magnetic resonance imaging, and coronary arteriography. Drug challenge with flecainide (Figure 1B) and epinephrine QT stress test were negative. 

**Figure 2:**
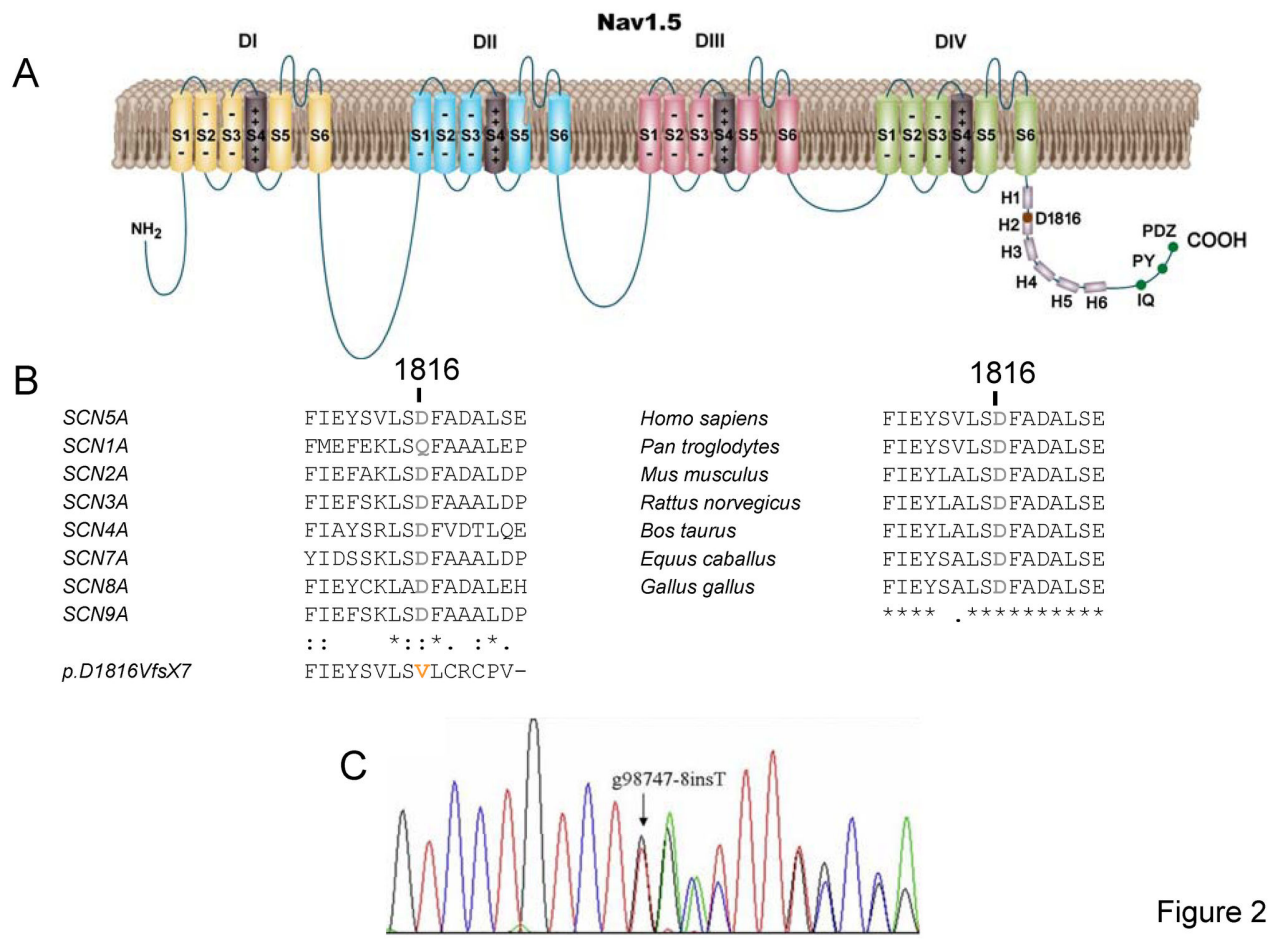
p.D1816VfsX7 mutation characteristics and localization. **A**. Topological diagram of Nav1.5 showing the location of D1816 residue. H1 to H6: each of the six α-helices present in the proximal half of the C-terminus. IQ: IQ motif which constitutes a calmodulin binding site. PY: PY motif which constitutes a binding site for E3 ubiquitin-protein ligases. PDZ: PDZ motif which constitutes a binding site for SAP97, PTPH1, and syntrophins. **B**. Sequence alignments of a segment of the C-terminus of *SCN5A* homologs in 7 different species and *SCNXA* family sodium channel expressed in human tissues. The sequence of p.D1816VfsX7 mutation is also shown. The respective accession numbers are NP_001159435.1, NP_001035232.1, NP_001075145.1, NP_000325.4, NP_000326.2, NP_002967.2, NP_001171455.1, and NP_002968.1 for SCN1-5/7-9, and for the different species are: XP_001171891.2 (*Pan troglodytes*), NP_001240789.1 (*Mus musculus*), NP_001153634.1 (*Rattus norvegicus*), NP_776883.1 (*Bos taurus*), NP_001157367.1 (*Equus caballus*), and XP_001232818.2 (*Gallus gallus*). “*” means that the residues in that column are identical in all the sequences in the alignment. “:”indicates conservation between groups of strongly similar properties. “.”indicates conservation between groups of weakly similar properties. **C**. DNA sequence chromatograms of the proband depicting the heterozigous insertion in exon 28 of *SCN5A*.

Proband`s genetic analysis identified a frameshift mutation consisting in a T insertion at position 98747 of *SCN5A* (exon 28) (Figure 2C) resulting in the substitution of the aspartic acid at position 1816 by a valine followed by 6 miscoded residues prior to a termination codon (p.D1816VfsX7) (Figure 2A and 2B). Thus, the mutation leads to the deletion of C-terminal 201 aminoacids of Nav1.5 protein. As shown in Figure 2B, this region which includes the D1816 residue, is highly conserved among human Nav1.x clones and Nav1.5 channels of different species. Importantly, the mutation was not observed in more than 200 control subjects. Moreover, sequence analysis of all other exons of *SCN5A* did not reveal any other mutation and screening for mutations in the other genes tested was also negative. D1816VfsX7 mutation has not been described either in the 1000 Genomes project or in the NHLBI Exome Sequencing Project.

**Table 1:** Comparison of ECG features between p.D1816VfsX7 mutation carriers and non carriers.

**ECG parameters**	**p.D1816VfsX7 non carriers**	**p.D1816VfsX7 carriers**
*N*	8	8
Spontaneous Brugada pattern	0	0
PR interval (ms)	150±29	197±27*
QRS interval (ms)	91±12	113±16*
QTc interval (ms)	392.3±9.0	398.7±6.1
Atrial fibrillation (%)	0 (0%)	2 (25%)

Values are the mean±SEM of 8 non carriers and 8 carriers in each case. * P<0.05 vs non carriers.

Genetic screening of fifteen family members revealed that seven of them also carried the mutation (Figure 1C). Electrocardiogram (ECG) recording, echocardiogram and flecainide tests were also performed on all the relatives studied. As can be observed in Table 1, PR and QRS intervals, but not QTc intervals, were significantly prolonged in the mutation carriers compared with non-carriers. Interestingly, the family screening detected an abnormal basal ECG in a son (24 years old; III:1) and a sister (59 years old; II:4) of the proband carrying the *SCN5A* mutation, with slightly widened QRS in V1-V3 and positive response with development of type 1 BrS pattern after flecainide continuous infusion at 2 mg/kg for 10 min (Figure 1D and Table 2). Another proband’s sister exhibited permanent AF (II:8). Moreover, another relative carrying the mutation (III:6) has presented a syncopal episode in the past (Table 2). The remaining relatives were all asymptomatic at the present time.

**Table 2:** Clinical phenotype of afected mutation carriers.

**Patient, (gender)**	**Clinical phenotype**
Proband, (F)	Syncope, Ventricular tachycardia and fibrillation, bradycardia and paroxysmal atrial fibrillation.
II:4 (F)	Abnormal basal ECG. Development of type 1 BrS after flecainide test.
II:8 (F)	Permanent atrial fibrillation
III:1 (M)	Abnormal basal ECG. Development of type 1 BrS after flecainide test.
III:6 (M)	Syncope.

BrS: Brugada Syndrome. ECG: Electrocardiogram, F: female, M: male.

### Functional characterization

**Figure 3:**
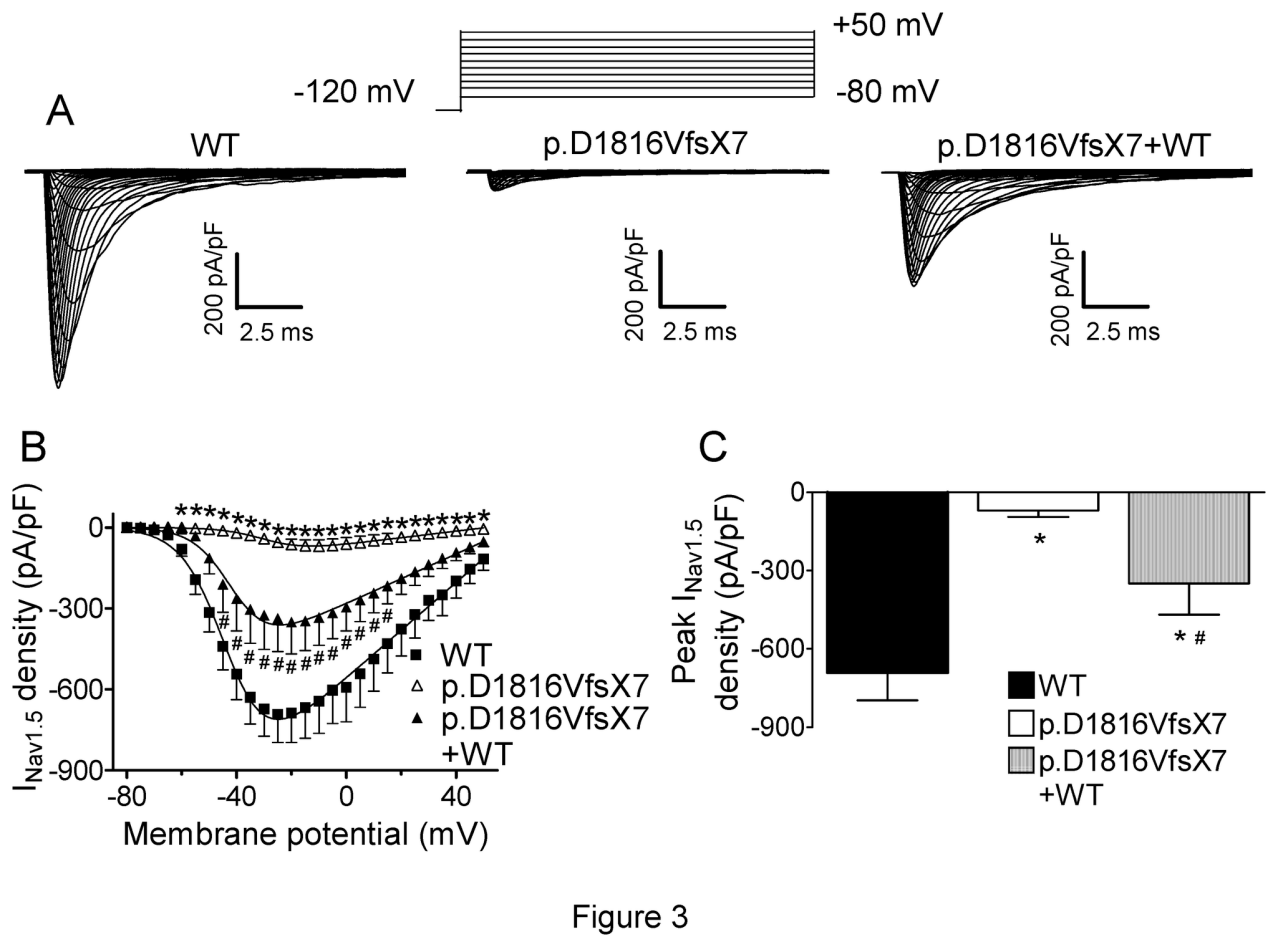
WT and p.D1816VfsX7 Nav1.5 current densities. **A**. Current traces obtained by applying the protocol shown at the top in cells transfected with WT, p.D1816VfsX7, and p.D1816VfsX7+WT. B. Current-density voltage relationships for WT, p.D1816VfsX7, and p.D1816VfsX7+WT channels. **C**. Peak current density generated by each construct tested. Each point/bar represents mean±SEM of >10 experiments. In B and C, *P<0.01 vs WT, and ^#^ P<0.01 vs p.D1816VfsX7.

**Figure 4:**
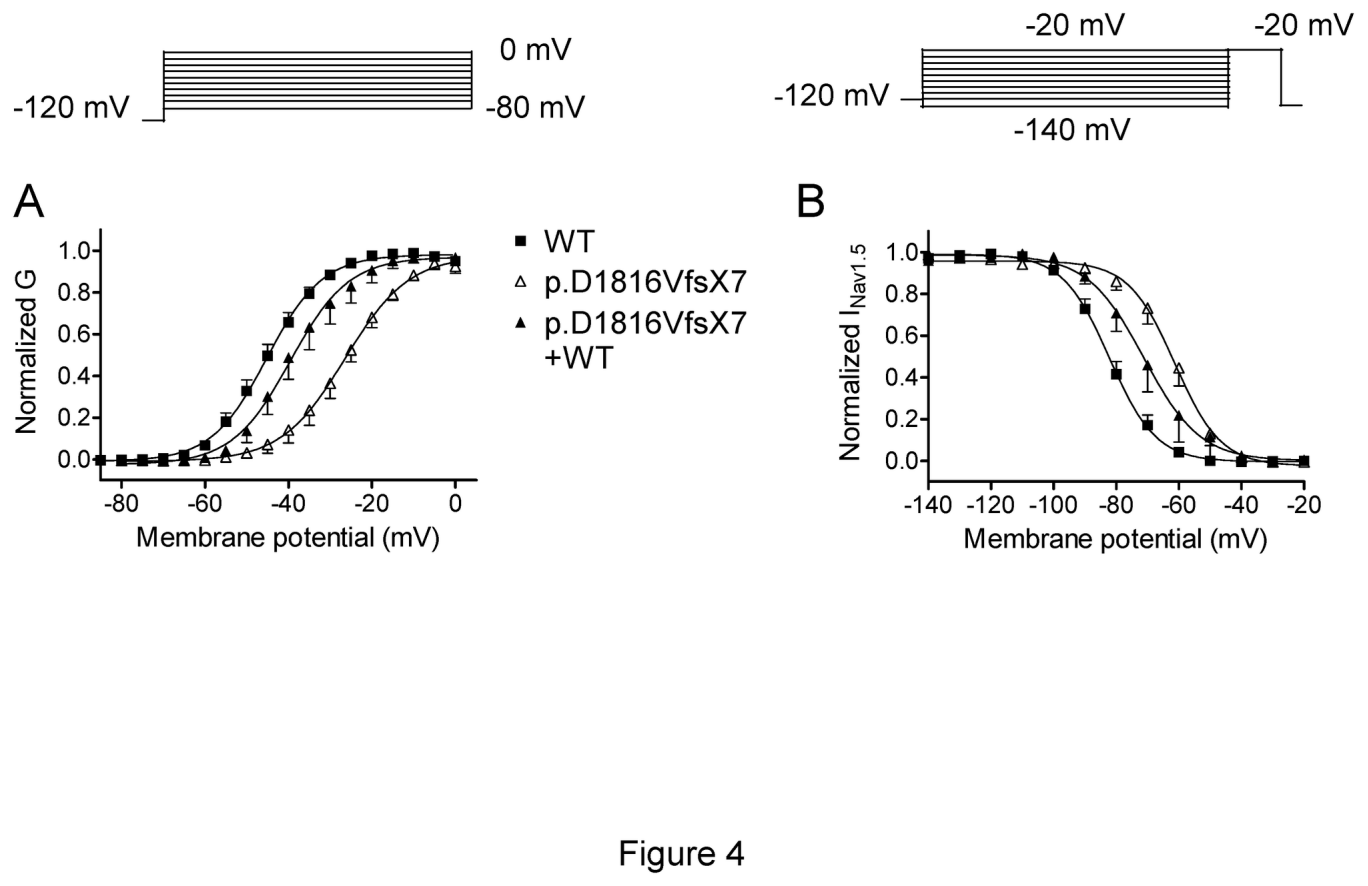
Activation and inactivation voltage-dependence of WT and p.D1816VfsX7 Nav1.5 channels. (**A** and **B**) Normalized steady-state activation (A) and inactivation curves (B) of WT, p.D1816VfsX7, and p.D1816VfsX7+WT constructs. Solid lines represent the Boltzmann fit to data point. Each point represents the mean±SEM of >10 experiments.

Figure 3A shows I_Nav1.5_ traces recorded by applying 50 ms pulses from -120 mV to potentials between -80 and +50 mV in cells expressing WT and p.D1816VfsX7 channels. Recent experimental evidence points to the existence of some kind of interaction between Nav1.5 subunits to form functional channels, in such a way that some trafficking deficient mutations produced dominant negative effects on WT channels [15,22]. Therefore, to mimic the heterozygous state of the patient and to determine whether the p.D1816VfsX7 mutation produced a dominant negative effect on WT channels, WT and p.D1816VfsX7 channels were cotransfected using a 0.5:0.5 ratio (p.D1816VfsX7+WT). Cells were always cotransfected with the gene encoding hNavβ1 subunit (1:1 ratio). Peak current density generated by p.D1816VfsX7 (-69.8±24.7 pA/pF) was dramatically smaller than that generated by WT channels (-691±105 pA/pF) (n>12; P<0.01) (Figures 3A-C). Peak current density generated by p.D1816VfsX7+WT channels (-350±119 pA/pF) was approximately 50% of the WT value (Figures 3A-C), a result which is consistent with haploinsuficiency without producing dominant negative effects. Interestingly, p.D1816VfsX7 peak current occurred at more positive potentials than that of WT channels (Figure 3B), suggesting that the mutation altered voltage dependence of activation. Indeed, as can be observed in Figure 4A and Table 3, p.D1816VfsX7 shifted ~18 mV positively the activation curve obtained by plotting the normalized peak conductance as a function of the membrane potential. Co-expression of WT with p.D1816VfsX7 mutation partially reversed this gating defect produced by the mutation (Figure 4A and Table 3). The effects of the mutation on the activation kinetics were assessed by measuring the time to peak. Interestingly, p.D1816VfsX7 significantly accelerated the time course of current activation, an effect that was completely abrogated when the mutant channel was cotransfected with WT (Table 3). Voltage dependence of steady-state inactivation was tested by applying 500 ms conditioning pulses from -120 to potentials between -140 and -20 mV followed by a test pulse to -20 mV. p.D1816VfsX7 mutation alone or in combination with WT channels shifted the midpoint of the inactivation curve to more positive potentials, without modifying the slope factor curve (Figure 4B and Table 3). Overall, these results suggested that p.D1816VfsX7 mutation profoundly affected the voltage dependence of Nav1.5 channels activation and inactivation. To rule out that these effects can be secondary to the presence of F^-^ ion in the intracellular solution, currents generated by WT and p.D1816VfsX7 channels were recorded in the absence of this ion. Importantly, the changes produced by the mutation were quantitatively and qualitatively identical when the currents were recorded in the absence of F^-^ in the intracellular solution (Table 4). 

**Table 3:** Effects of p.D1816VfsX7 mutation on time-, and voltage-dependent Nav1.5 channel properties.

**Nav1.5 construct**	**Activation**			**Inactivation**				**Reactivation**	
	**V_h_ (mV)**	***k* (mV)**	**Time to peak (ms)**	**V_h_ (mV)**	***k* (mV)**	**τ_f_ (ms)**	**τ_s_ (ms)**	**τ_f_ (ms)**	**τ_s_ (ms)**
**WT**	-45.2±1.4	5.6±0.4	0.53±0.03	-82.1±1.9	6.5±0.3	0.9±0.1	5.7±0.3	3.5±0.6	18.8±2.4
**p.D1816VfsX7**	-27.3±2.1*	5.4±0.4	0.28±0.02*	-61.5±2.5*	6.0±1.0	0.8±0.1	4.7±0.6	1.5±0.3*	24.5±3.4
**p.D1816VfsX7+WT**	-38.5±6.6* ^#^	5.5±0.5	0.54±0.06	-69.6±6.5* ^#^	6.1±0.4	1.1±0.1	5.8±1.0	2.2±0.4* ^#^	17.9±4.0

V_h_ and *k*: midpoint and slope values of the activation and inactivation curves. τ_f_ and τ_s_: fast and slow time constants of inactivation or reactivation

Values are mean±SEM of >10 experiments. * P<0.01 vs WT. ^#^ P<0.01 vs p.D1816VfsX7

**Table 4:** Comparison of voltage-dependence of activation and inactivation parameters from the currents generated by WT and mutant Nav1.5 recorded in the absence of F^-^.

**Nav1.5 construct**	**Activation**			**Inactivation**			
	**V_h_ (mV)**	***k* (mV)**	**Time to peak (ms)**	**V_h_ (mV)**	***k* (mV)**	**τ_f_ (ms)**	**τ_s_ (ms)**
**WT**	-33.8±1.4	5.2±0.9	0.4±0.06	-72.0±4.3	6.9±0.4	0.9±0.1	9.1±1.6
**p.D1816VfsX7**	-12.6±1.8**	6.2±0.6	0.1±0.02**	-55.3±2.3**	6.4±0.6	0.9±0.1	10.4±1.6

V_h_ and *k*, midpoint and slope of the activation and inactivation curves. τ_f_ and τ_s_, fast and slow time constants of inactivation. Values are the mean±SEM of 10 experiments in each group. ** P<0.01 vs WT.

**Figure 5:**
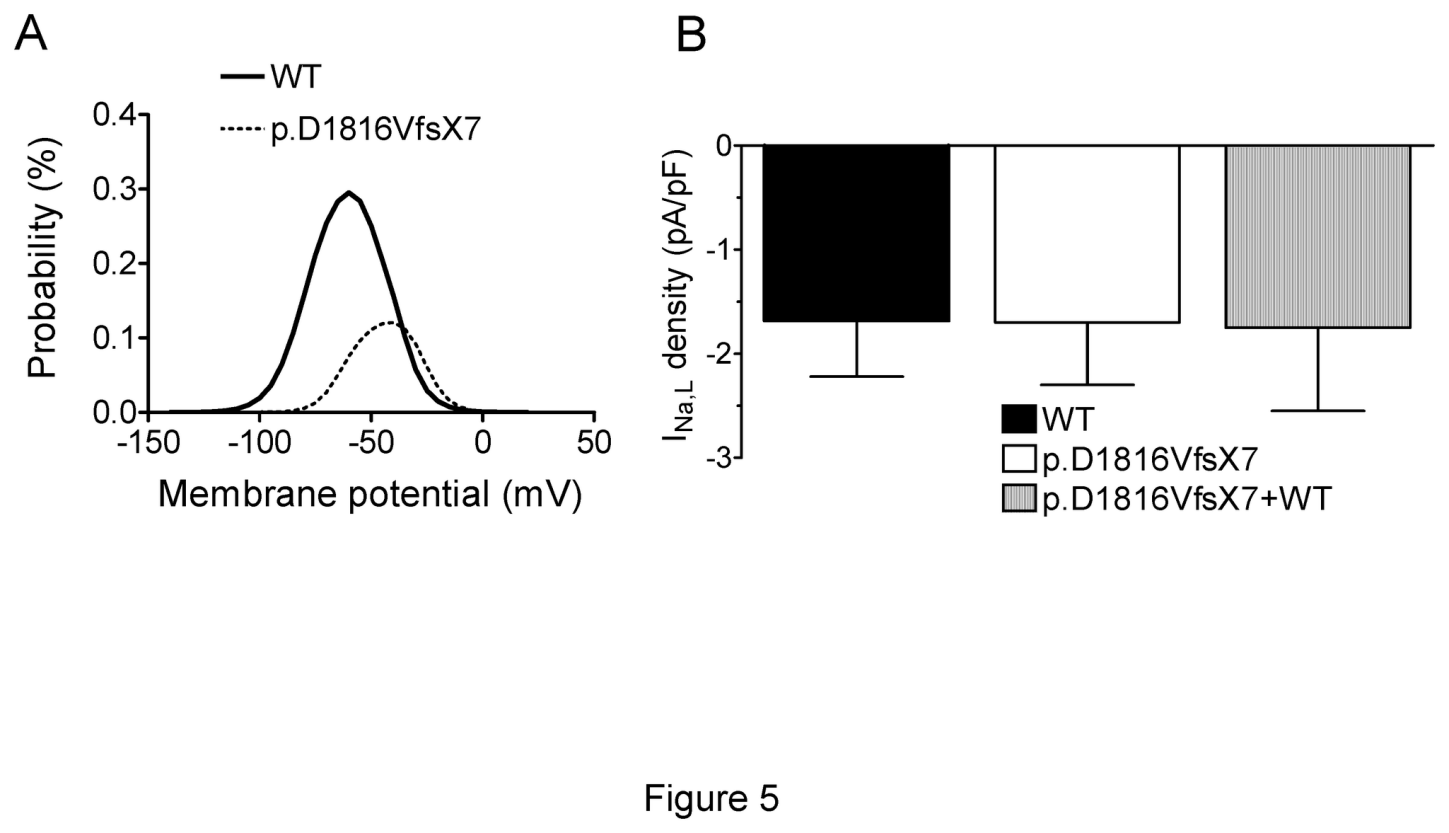
Effects of p.D1816VfsX7 mutation on the window and late Na^+^ currents. **A**. Effects of p.D1816VfsX7 mutation on the window current. The probability of being within this window for WT and p.D1816VfsX7 channels is plotted. **B**. Effects of the mutation on the I_NaL_. Bar graphs show the density of the current generated by WT, p.D1816VfsX7, and p.D1816VfsX7+WT constructs measured at the end of 500-ms pulse to -20 mV from a holding potential of -120 mV. Each bar represents the mean±SEM of >12 experiments.

We also determined the effects of the mutation on the window current. The overlap of the activation and steady-state inactivation of Na^+^ channels identifies a range of voltages (i.e., window) where the channels have a small probability of being partially but not fully inactivated. The plot of the probability of being within the window calculated from the product of the fitted activation and steady-state inactivation parameters (Figure 5A) demonstrated that p.D1816VfsX7 decreased the amplitude of the window current and shifted its peak to more depolarized potentials (from 0.29±0.1% at -60 mV for WT channels to 0.12±0.04% at -40 mV for p.D1816VfsX7 channels; P<0.05). 

To determine putative modifications on the kinetics of the fast inactivation process, a biexponential function was fitted to the decay of the peak current traces elicited by each construct. Interestingly, both fast and slow time constants of inactivation of p.D1816VfsX7 were indistinguishable from those of WT channels (Table 3), demonstrating that the mutation did not modify fast inactivation kinetics. Furthermore, the mutation did not modify the current density after 500 ms pulses to -20 mV, indicating that the density of the I_NaL_ was not changed by the mutation (Figure 5B).

**Figure 6:**
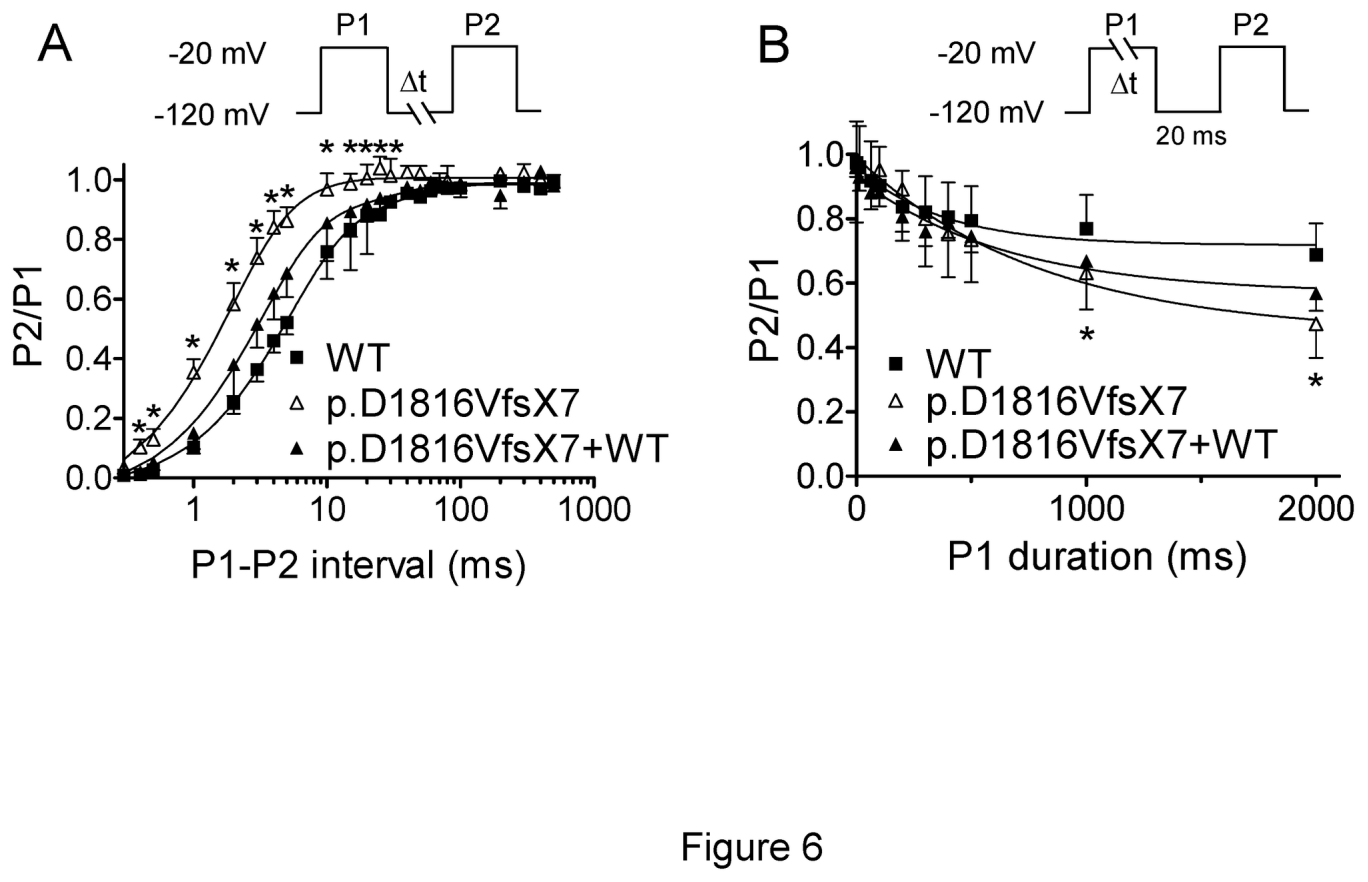
Recovery from fast inactivation of WT and p.D1816VfsX7 Nav1.5 channels. **A**. Recovery from fast inactivation of WT, p.D1816VfsX7, and p.D1816VfsX7+WT constructs assessed by applying two 500 ms pulses from -120 to -20 mV (P1 and P2) at increasing coupling intervals (0.1–500ms). Solid lines represent the fit of a biexponential function to the data points. **B**. Development of slow inactivation of WT, p.D1816VfsX7, and p.D1816VfsX7+WT constructs assessed with the double-pulse protocol shown at the top. Solid lines represent the fit of a monoexponential function to the data points. Each point (ratio of the peak current amplitude generated by P2 and P1 pulses) represents the mean±SEM of >10 experiments and * P<0.05 vs WT.

Recovery from fast inactivation was analyzed by applying two test pulses to -20 mV with increasing coupling intervals (Figure 6A). The holding and the potential between pulses were -120 mV. Recovery time course was depicted by biexponential functions yielding the fast and slow time constants reported in Table 3. Interestingly, recovery from fast inactivation of p.D1816VfsX7 channels was significantly faster than those of WT and p.D1816VfsX7+WT channels (Figure 6A and Table 3). We next analyzed the development of slow inactivation by applying a pre-pulse to -20 mV of progressively increasing duration followed by a test pulse also to -20 mV. p.D1816VfsX7 mutation significantly increased the fraction of channels that developed slow inactivation with prolonged depolarizations (Figure 6B). Co-expression of p.D1816VfsX7 with WT channels partially reversed this alteration. 

### Effects of p.D1816VfsX7 mutation on channel trafficking

**Figure 7:**
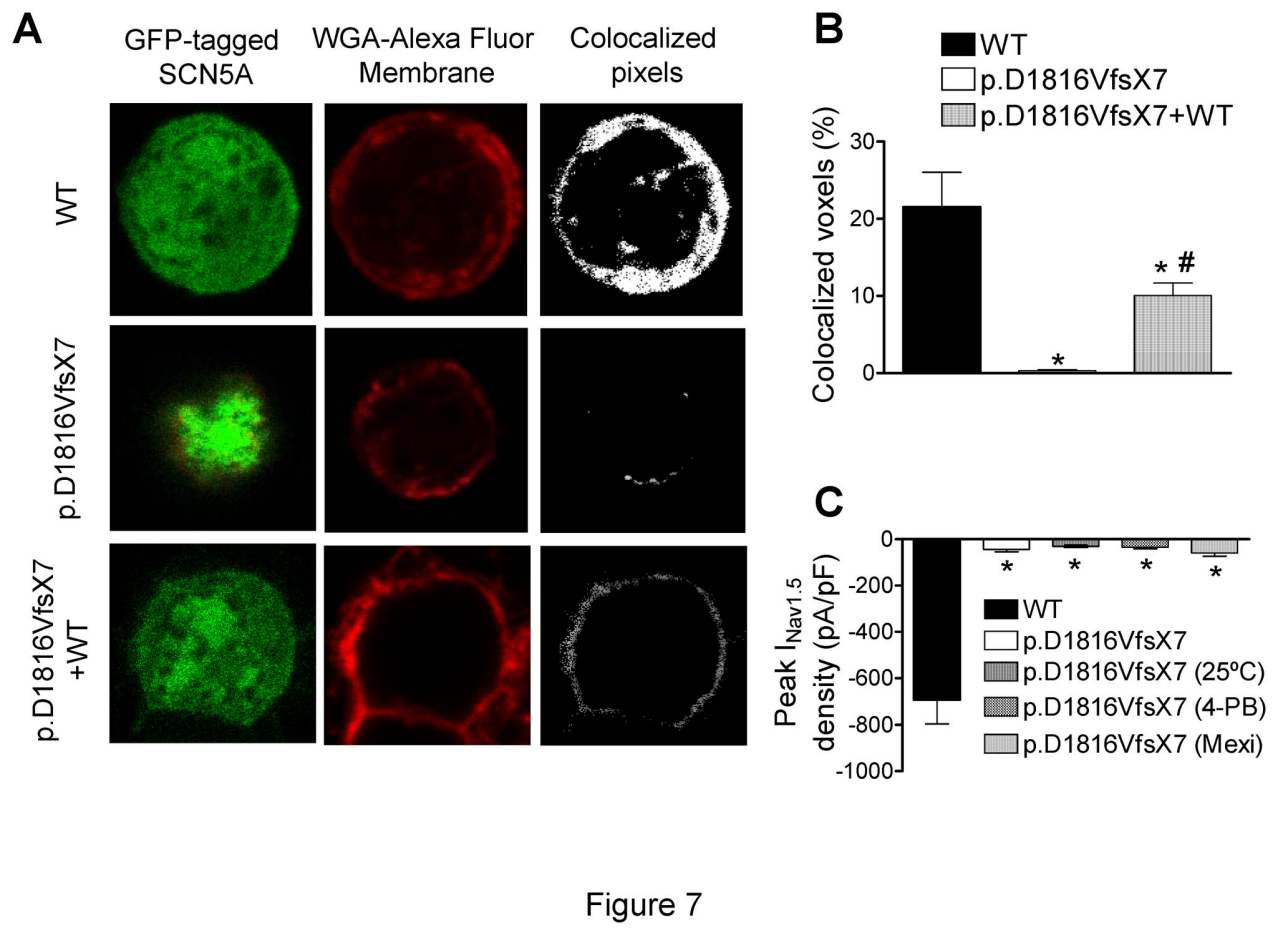
WT and p.D1816VfsX7 Nav1.5 channel trafficking. **A**. Representative single fluorescent confocal images from the center of CHO cells transfected with GFP-tagged-Nav1.5 WT, p.D1816VfsX7, and p.D1816VfsX7+WT constructs. Columns: GFP-tagged-Nav1.5 channel staining (left), wheat germ agglutinin Alexa Fluor 647 (a fluorescent membrane dye) staining (middle), and colocalization of both (right). **B**. Percentage of colocalized voxels measured in cells transfected with WT, p.D1816VfsX7, and p.D1816VfsX7+WT. **C**. Comparison of peak current-density recorded in cells expressing p.D1816VfsX7 incubated or not at 25°C, or in the presence of 5 mM 4-phenylbutirate (4-PB) or 300 μM mexiletine (mexi) with WT. Bars represent the mean±SEM of >6 cells. * P<0.05 vs WT and ^#^ P<0.05 vs p.D1816VfsX7.

In the next group of experiments, the effects of p.D1816VfsX7 mutation on Nav1.5 trafficking using channels conjugated with GFP were analyzed by using confocal microscopy. Additionally, we performed a group of experiments where we compared the currents generated by Nav1.5 WT channels conjugated or not with GFP. As is shown in Table 5, under our experimental conditions, current density, time-dependent kinetics and voltage dependence of activation and inactivation were not significantly different, confirming that the presence of GFP does not interfere with the electrophysiological properties of the channel. On the other hand, the confocal microscopy results obtained demonstrated that WT channels were distributed uniformly throughout the cell including the cell membrane as demonstrated by the colocalization of the GFP signal with that of the membrane marker wheat germ agglutinin Alexa Fluor 647 (Figure 7A upper panel). On the contrary, cells expressing p.D1816VfsX7 channels did not clearly exhibit fluorescence in the membrane as the GFP signal was mainly localized in the cytoplasm and, thus, colocalization was significantly decreased compared with WT channels (Figure 7A middle panel and 7B). Membrane fluorescence significantly increased when p.D1816VfsX7 channels were coexpressed with WT channels (Figure 7A and B).

**Table 5:** Comparison of voltage-dependence of activation and inactivation parameters between Nav1.5 conjugated or not with GFP.

**Nav1.5 construct**	**Activation**			**Inactivation**			
	**V_h_ (mV)**	***k* (mV)**	**Time to peak (ms)**	**V_h_ (mV)**	***k* (mV)**	**τ_f_ (ms)**	**τ_s_ (ms)**
**WT**	-38.8±2.6	6.1±0.9	0.5±0.2	-81.7±6.0	6.1±0.4	0.9±0.2	6.3±0.7
**WT-GFP**	-38.5±2.4	6.4±0.7	0.4±0.2	-79.2±5.5	7.1±0.8	1.1±0.8	6.2±2.0

V_h_ and *k*, midpoint and slope of the activation and inactivation curves. τ_f_ and τ_s_, fast and slow time constants of inactivation. Values are the mean±SEM of 10 experiments in each group.

Previous data have demonstrated that trafficking of Nav1.5 mutant channels can be restored by decreasing incubation temperature during cell culture or by incubation with compounds or drugs (Class I antiarrhythmic drugs) that improve channel trafficking [23]. Reducing the incubation temperature during cell culture to 25°C or incubation of the p.D1816VfsX7 transfected cells with 5 mM 4-phenylbutirate or 300 μM mexiletine for 48 h did not modify current density compared to that recorded under control conditions (Figure 7C).

## Discussion

In the present study, we describe the functional consequences of a frameshift mutation (p.D1816VfsX7) found in a patient with repeated episodes of ventricular fibrillation who lacks typical ECG features of BrS or long QT syndrome both under basal conditions and after flecainide and epinephrine challenges. There are previous reports demonstrating that loss-of-function SCN5A mutations could result in syncope, ventricular fibrillation, and sudden cardiac death in patients with normal structural hearts who do not exhibit any electrocardiographic manifestations of inherited arrhythmogenic syndromes [6,7]. However, it is important to note that the proband presented sinus bradycardia on basal status and under flecainide, which could be attributed to the p.D1816VfsX7 mutation. This hypothesis could seem counterintuitive since pacemaker activity in sinus nodal cells is mainly regulated by Ca^2+^ and K^+^ channels. However, bradyarrhythmic complications have been associated with other Nav1.5 loss-of-function mutations [24,25]. Indeed, it has been described that Nav1.5 channels are distributed in the periphery of the sinus node [26], and thus, it is possible that a functional defect of Nav1.5 channel activity would lead to reduced impulse propagation from the sinoatrial node to the surrounding atrial muscle and, thus, to a decrease in heart rhythm [24,25].

Genetic screening of several family members revealed that seven of them also carry the mutation. Among them other cardiac electrical abnormalities were detected. As mentioned, the proband and one of her sisters exhibit paroxysmal and permanent AF, respectively. A son and a sister of the proband displayed type 1 BrS pattern after flecainide challenge. Moreover, another relative carrying the mutation had suffered an episode of syncope in the past. Additionally, PR and QRS intervals were significantly prolonged in the mutation carriers. Although the conduction delay is mild the concomitant presence of both PR and QRS prolongation suggests a hampered conduction in different cardiac compartments. Our hypothesis is that impairment of intracardiac conduction exhibited by mutant carriers and the BrS exhibited by two of them, could be attributed to the marked I_Na_ decrease produced by the mutation. Furthermore, Nav1.5 loss-of-function mutations have been associated with atrial arrhythmias including AF [4]. In fact, prevalence of AF and atrial flutter in patients with BrS is very high (approximately 20%) [27]. Moreover, it has been suggested that some patients with drug-induced BrS ECG may develop atrial arrhythmias prior to developing ventricular fibrillation [27]. Therefore, we propose that this severe loss-of-function mutation is associated with incomplete penetrance and variable expressivity giving rise to a complex phenotype which includes ventricular fibrillation, bradycardia, intracardiac conduction delay, AF, and BrS. The association of a single *SCN5A* mutation with a wide spectrum of disease phenotypes has been described previously [8]. Unfortunately, the reasons underlying the marked heterogenity in phenotype remain unknown. Gender and age may constitute clinical determinants of disease expressivity. However, in our family three sisters of similar age clearly differ in their phenotype, i.e., ventricular fibrillation associated with bradycardia and paroxysmal AF (the proband) vs. permanent AF (II:8), and BrS (II:4). Moreover, epigenetic or genetic factors including genetic variability due to the presence of polymorphisms, may contribute to disease expressivity and mixed phenotypes in Nav1.5 loss-of-function overlap syndromes [8]. Therefore, unknown genetic or environmental factors may probably account for the variable and incomplete disease expressivity in this family. 

p.D1816VfsX7 mutation produces a 201-residue truncation of the C-terminal domain of the channel that markedly affected channel expression and gating. Indeed, the most important alteration of Na^+^ channel function produced by the mutation was a dramatic reduction (≈90%) in peak current density as a consequence of a significant decrease of channel trafficking toward the membrane. Furthermore, impaired channel trafficking was not restored by incubating transfected cells at low temperature or with chemical chaperones such as mexiletine or 4-phenylbutirate. Trafficking defects of mutant Na^+^ channel proteins can be due to endoplasmic reticulum retention, degradation of folding-deficient proteins or absence of a motif essential for trafficking [28]. The C-terminal domain of Nav1.5 channels, comprising residues 1773 to 2016, contains several protein-protein interaction motifs including a calmodulin-binding IQ motif (residue 1908), a PY motif (residue 1974) and a PDZ binding domain motif (3 last residues) (Figure 2). It has been described that interaction of calmodulin, ubiquitin protein ligases and PDZ domain-bearing proteins with these domains is needed for proper expression of the channel [29,30]. Especially important is the interaction of Nav1.5 with PDZ domain-bearing proteins such as the protein tyrosine phosphatase (PTPH1), the membrane associated guanylate kinase scaffolding protein (SAP97), and syntrophin-dystrophin complex. These proteins recognize the C-terminal Ser-Ile-Val (SIV) sequence of Nav1.5 and subsequently increase channel expression in the membrane [29,30]. p.D1816VfsX7 mutant channel lacks all these protein-protein interaction motifs, a fact that provides a plausible mechanism to explain its trafficking defect.

**Figure 8:**
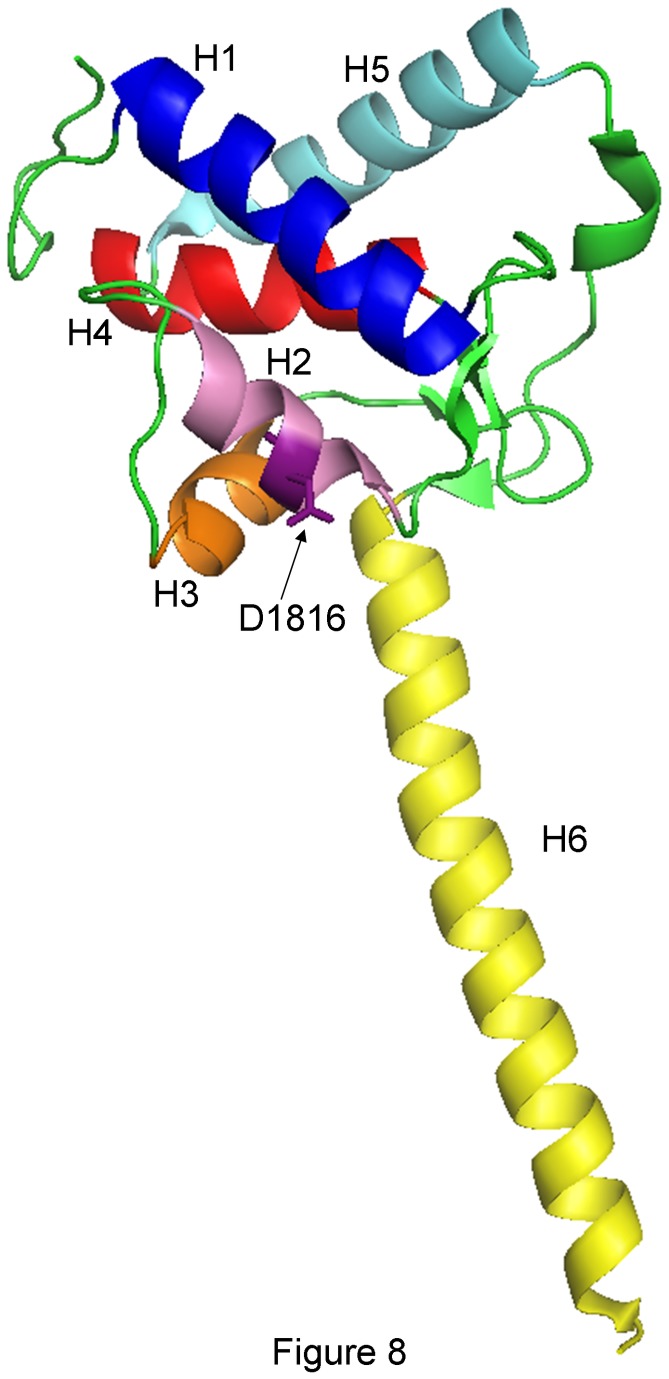
Crystalized structure of the proximal part of Nav1.5 C-terminus (PDB 4DCK). H1 to H6 helical structures as well as the location of D1816 have been highlighted. H1 (blue); H2 (pink); H3 (orange); H4 (red); H5 (light blue); H6 (yellow); D1816 (deep purple).

It is interesting to note that despite the severe truncation of the C-terminus of Nav1.5, p.D1816VfsX7 channels generated measurable but abnormal currents. The mutation induced alterations in channel gating leading to a mixed loss- and gain-of-function phenotype. Indeed, it produced a positive shift in the voltage dependence of activation and an increase in the fraction of channels entering the slow inactivated state, effects that would reduce peak I_Nav1.5_. On the contrary, it positively shifted the voltage dependence of fast inactivation and accelerated activation and recovery from fast inactivation kinetics, effects that would contribute to an increase in channel availability. Interestingly, the mutation markedly reduced and shifted the peak of the window current. The window current determines a range of membrane potentials where Na^+^ channels can be activated but not inactivated. p.D1816VfsX7 mutation would lead to channel opening at more depolarized potentials and, thus, to a further decrease in cell excitability. However, the mutation did not modify I_NaL_ which correlates with the lack of QT prolongation in any of the mutant carriers. Other frameshift mutations leading to the truncation of the C-terminus have been described (p.L1786EfsX2, p.F1808IfsX3, p.E1823Hfs10, and p.R1860KfsX13 associated with BrS and p.L1821fsX10 associated with sick sinus syndrome, cardiac conduction disease and ventricular tachycardia) [11,31]. Among these mutations only p.L1821fsX10 was functionally studied and its expression in HEK-293 cells demonstrated that the mutation also generated currents, albeit dramatically reduced [11]. Conversely, complete truncation of the C-terminal domain leads to non detectable currents [32]. Thus, to our knowledge, p.D1816VfsX7 produces the greater disruption of Nav1.5 protein sequence for a disease causing mutation that still generates measurable currents. Some of the effects of p.L1821fsX10 on channel gating (shift of the activation curve to more positive potentials, enhanced slow inactivation) were similar to those produced by p.D1816VfsX7 [11]. However, other effects of the p.L1821fsX10 mutation (shift of the inactivation curve to negative potentials and increased I_NaL_ density) were not shared by p.D1816VfsX7 mutation. It has been described that the C-terminal cytoplasmic domain, is important for setting the properties of fast inactivation [9,32]. Thus, it can be somewhat surprising that two frameshift mutations p.D1816VfsX7 and p.L1821fsX10 that lead to similar deletions of the C-terminus (201 and 195 residues, respectively) can differentially affect voltage dependence of fast inactivation or I_NaL_. The proximal half of the C-terminus contains six helical structures (H1-H6) (Figure 8) that are critical for establishing interhelical contacts as well as intramolecular interactions with domain III and IV linker that stabilize the inactivated state [10,33,34]. Interestingly, p.L1821fsX10 truncation removes H3 to H6 but leaves H1 and H2 completely unaltered. However, p.D1816VfsX7 that also truncated H2 (Figure 8), may impair some other interactions different from those disrupted by p.L1821fsX10. Therefore, our results confirm that C-terminal regions of Na^+^ channel mutants may contribute to differences in gating properties of the variants. Furthermore, our results confirm the important role of the C-terminus for Nav1.5 channel expression and function and demonstrate that the length of the truncated C-terminus segment may have important implications for Na^+^ channel function.
